# Acupressure in patients with seasonal allergic rhinitis: a randomized controlled exploratory trial

**DOI:** 10.1186/s13020-021-00536-w

**Published:** 2021-12-18

**Authors:** Lukas Israel, Gabriele Rotter, Ulrike Förster-Ruhrmann, Josef Hummelsberger, Rainer Nögel, Andreas Michalsen, Tatjana Tissen-Diabaté, Sylvia Binting, Thomas Reinhold, Miriam Ortiz, Benno Brinkhaus

**Affiliations:** 1 Institute of Social Medicine, Epidemiology, and Health Economics, Charité – Universitätsmedizin Berlin, Freie Universität Berlin and Humboldt-Universität zu Berlin, Luisenstr. 57, 10117 Berlin, Germany; 2 Department for Otolaryngology, Charité – Universitätsmedizin Berlin, Freie Universität Berlin and Humboldt-Universität zu Berlin, Charité Platz 1, 10117 Berlin, Germany; 3 Societas Medicinae Sinensis (SMS) e.V. - International Society for Chinese Medicine, Franz-Joseph-Straße 38, 80801 Munich, Germany

**Keywords:** Acupressure, Chinese medicine, Rhinitis quality of life, Seasonal allergic rhinitis

## Abstract

**Background:**

Acupuncture has shown beneficial effects for seasonal allergic rhinitis (SAR); however, it is time and cost intensive. We investigated feasibility and effects of self-administered body acupressure as a self-care technique that stimulates acupuncture points with manual pressure in SAR patients.

**Methods:**

We conducted a two-armed randomized controlled exploratory trial to compare effects of self-administered acupressure over 4 weeks at five acupuncture points plus rescue medication (RM) with cetirizine compared to RM alone in SAR patients. Among other outcome parameters, we assessed disease-related quality of life (Rhinitis Quality of Life Questionnaire [RQLQ]), overall SAR symptoms by a visual analogue scale (VAS) and a rescue medication score (RMS) after 4 and 8 weeks.

**Results:**

Forty-one SAR patients (mean age 38.5 ± 10.0 years, n = 21, 51.2% women) were randomized. Compared to RM alone (n = 21), acupressure plus RM (n = 20) was associated with relevant improvements after 4 weeks, shown by the difference between groups in adjusted means of RQLQ: − 0.9 points (95% CI − 1.6 to − 0.2; p = 0.011) and VAS overall SAR symptoms: − 21.6 mm (95% CI − 36.3 to − 6.8; p = 0.005). The RMS was lower in the acupressure group than in the control group: 1.9 points (95% CI − 3.8 to − 0.1; p = 0.120). Group differences decreased slightly until week 8. The acupressure was feasible and safe.

**Conclusion:**

Results of this exploratory study indicate that self-applied acupressure is feasible, may improve disease-specific quality of life and reduce disease-related symptoms as well as anti-allergic medication intake in SAR patients. High-quality confirmatory studies including a sham-control group are needed in the future.

*Trial registration* DRKS-ID: DRKS00014310. Date of registration in DRKS: 2018/04/24. Investigator sponsored/initiated trial (IST/IIT): yes. Ethics approval/approval of the ethics committee: Approved (leading) Ethics Committee No. EA1/033/18, Ethik-Kommission der Charité -Universitätsmedizin Berlin. URL: https://www.drks.de/drks_web/navigate.do?navigationId=trial.HTML&TRIAL_ID=DRKS00014310

**Supplementary Information:**

The online version contains supplementary material available at 10.1186/s13020-021-00536-w.

## Background

Allergic rhinitis, in its intermittent and perennial forms (traditionally termed seasonal and perennial) is a highly prevalent disease [1] that affects up to 30% of Europeans [2] and 12–30% of US Americans [3]. It is a high-cost medical condition that, in the United States, results in expenditures of a minimum of $11.2 billion US annually [4]. Although prevention strategies have been defined [5] and consensus therapy guidelines have been implemented [6, 7], many patients fail to obtain full symptom relief [8], and up to 20% of individuals with allergic rhinitis remain highly impaired [9]. Therefore, many patients use complementary medicine treatments, such as acupuncture, to improve seasonal allergic rhinitis (SAR) symptoms, and acupuncture is often used in Germany (17% lifetime prevalence) for SAR conditions [10, 11]. Previous trials have shown that acupuncture can lead to improvements in disease-specific quality of life by reducing SAR symptoms as well as a reduction in anti-allergic medication [12, 13]. Therefore, acupuncture has been recommended as an optional treatment in the new clinical practice guidelines developed by the American Academy of Otolaryngology [7].

Similarly, acupressure has been a legitimate component of Chinese medicine (CM) since its inception and represents a non-invasive manipulation technique in which manual pressure is used to stimulate acupuncture points along meridians on the body or ear [14]. Compared to massage therapy (MT) which is applied on less specific parts of the body to soften tissue and reduce pain, acupressure within the practice of CM stimulates biologically active points and can help reduce concentrations of stress hormones [15, 16]. Acupressure has shown therapeutic effects for patients with perennial allergic rhinitis (ear acupressure), chemotherapy-induced nausea and vomiting, primary dysmenorrhoea and cancer-related fatigue as well as for the induction of labour (body acupressure) [17–21]. A systematic review of four randomized controlled trials (RCTs) (n = 160) in patients with allergic rhinitis or asthma suggested that acupressure leads to better symptom alleviation than 1% ephedrine nasal drops plus thermal therapy [22].

Because acupuncture has shown positive effects for SAR, we hypothesized that self-administered acupressure represents a potential therapeutic and cost-effective option for SAR patients. Therefore, the aim of the study was to investigate the feasibility and effects of self-administered acupressure of the body (acupressure) in patients with SAR.

## Methods

### Design

In this two-armed, controlled exploratory prospective trial, we randomized SAR patients to an acupressure plus rescue medication (RM) group (acupressure group) or RM alone group (control group). In the acupressure group, acupressure was self-administered daily over 4 weeks, in addition to RM. After 4 weeks, patients could opt to continue the acupressure until the end of week 8. The control group received RM alone. All patients received follow-up until week 8. After completing the 8-week study period, the control group was given the option to receive acupressure training (waiting list design) (Additional file 1).

The trial was conducted at the Charité outpatient clinic for Complementary and Integrative Medicine in Berlin during the birch and grass pollen season in 2018 and in 2019. All study patients were informed individually about the study by the study physician, and they provided written informed consent. After completing the trial, all patients received 30€ as reimbursement for costs due to study participation (e.g., travel expenses).

To generate the randomization schedule, we used SAS 9.4 (Copyright 2002–2012 by SAS Institute Inc., Cary, NC, USA). Patients were registered and then randomized in a 1:1 allocation ratio by a computer-generated randomization process in the study centre. The allocation was performed in the study centre by a study nurse at Charité – Universitätsmedizin, Berlin and was concealed. The study physician was informed about the randomization result using consecutive numbering codes for each patient. Personal data were saved in a Microsoft Access database.

### Study patients

Patients were recruited mainly by subway advertisements, digital media, posters and flyers at the University Campus of the Charité in Berlin Mitte. The inclusion criteria were as follows: age 18 to 60 years; a diagnosis of SAR with a symptom duration of at least two years; IgE positivity to grass and birch pollen determined by either a skin-prick test or a RAST-resp. CAP-test; moderate to severe SAR symptom severity during the previous year and during the last 7 days, each defined as symptom severity rated between 30 and 70 mm on a visual analogue scale (VAS, 0–100 mm, 0 = no symptoms 100 = worst/maximal symptoms); and indication for oral intake of antihistamines and/or cortisone as anti-allergic treatment. The main exclusion criteria were perennial AR, chronic rhinosinusitis, allergic asthma and/or moderate to severe atopic dermatitis, other pulmonary diseases, autoimmune disorders whose symptoms resembled SAR, severe acute and/or chronic diseases, specific immunotherapy during the duration of the study, pregnancy or breastfeeding and acupuncture treatment or the use of any complementary medicine for SAR during the duration of the study.

### Study intervention acupressure

Following a modified consensus Delphi approach, four experienced CM experts from two German medical acupuncture societies developed the standardized acupressure procedure for this trial. This included the choice of five acupressure points acupoints LI-4, LI-11, LI-20, Gb-20 and Ex-HN 3 (Yintang) as well as the duration, frequency and intensity of acupressure. According to CM theories, all five points represent effective tools to treat SAR. LI-4 (Hegu) is essential to move Qi and improve blood circulation and, like other acupoints, it expels wind which includes allergic diseases such as SAR in Western medicine [23]. It has been shown that LI-4 may lead to improvement of respiratory function [24]. LI-11 clears heat, reduces itch and is known for its immune modulating and anti-inflammatory effects [23]. LI-20 liberates the nose and lungs [23] and may have, like Ex-HN 3 (Yintang), positive effects on pathological airway remodelling [25]. Ex-HN 3 (Yintang) reduces wind and is used for nasal discomfort such as allergic rhinitis, obstruction and sinusitis [23]. Gb-20 distributes wind, clears heat and is used for all forms of headache as well as infections of the upper airway [23]. It may have analgesic effects by decreasing the number of mast cells and macrophages [26]. Each patient assigned to the acupressure group received individual acupressure training lasting 20–30 min. This included an introduction to CM and a thorough demonstration of the acupressure points and technique, including the exact pressure to apply. In addition, detailed instructions in written form and video for domestic use were given by a study physician qualified in CM. During the 4-week acupressure period, patients were required to apply acupressure daily at all five determined points for a minimum of 20 min per day. They were permitted to choose either two daily sessions of 10 min or one session of 20 min (Table 1).

**Table 1:** Acupressure points and application modes

Point	Mode 1		Mode 2
	Session 1 (10 min)	Session 2 (10 min)	One session (20 min)
Ex-HN 3 (*Yintang*)	2 min	2 min	4 min
LI-4 (*Hegu*)	*Right* hand 2 min	*Left* hand 2 min	*Each side* 2 min
LI-11 (*Quchi*)	*Right* arm 2 min	*Left* arm 2 min	*Each side* 2 min
LI-20 (*Yingxiang*)	Bilateral 2 min	Bilateral 2 min	Bilateral 4 min
Gb-20 (*Fengchi*)	Bilateral 2 min	Bilateral 2 min	Bilateral 4 min

Ex-HN 3: extraordinary point 3 (hall of impressions)

LI-20: large intestine 20 (receiving fragrance)

LI-11: large intestine 11 (pool at the bend)

LI-4: large intestine 4 (junction valley)

Gb-20: gallbladder 20 (wind pool)

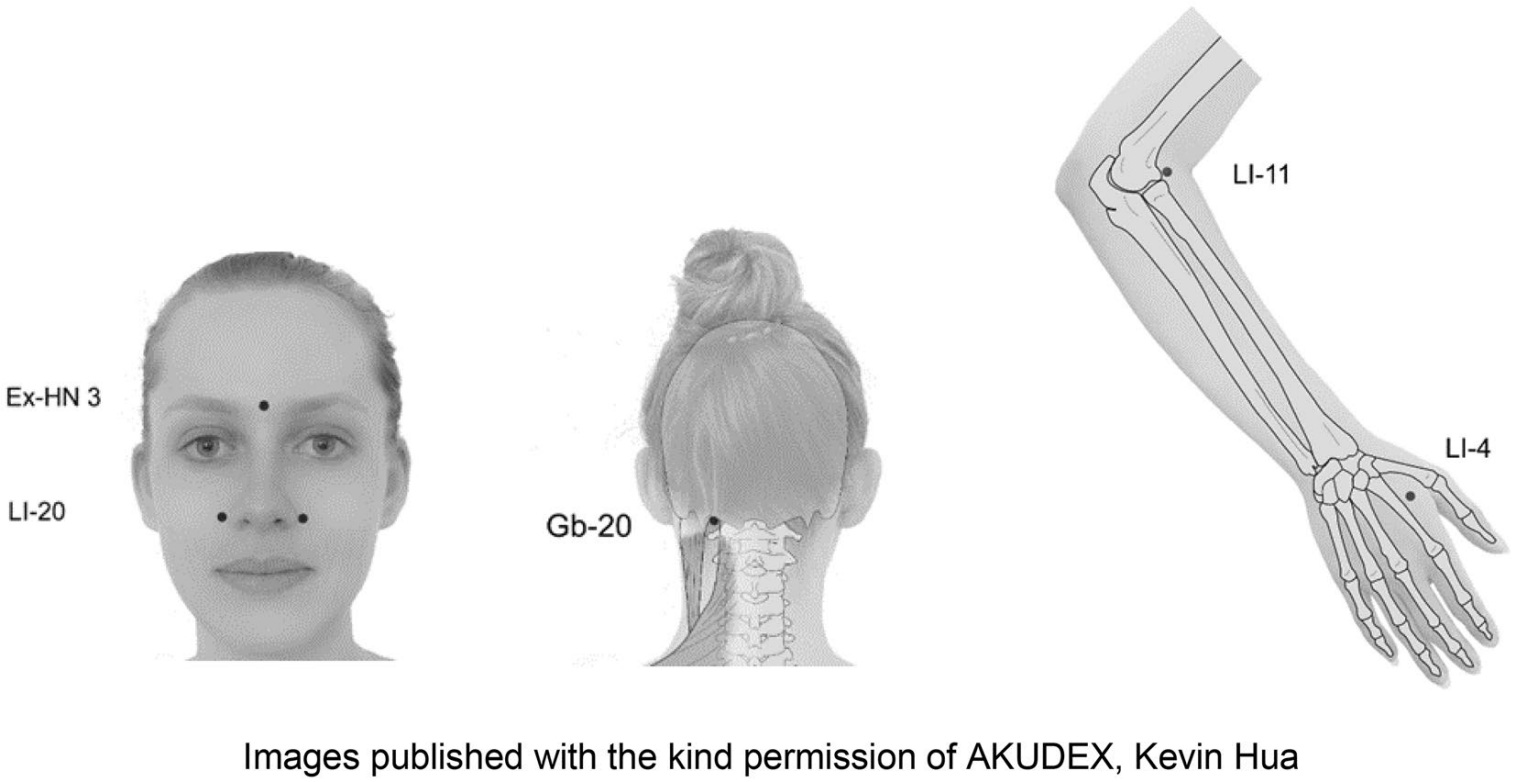

Oral cetirizine 10 mg (maximum two times daily) and an oral corticoid (prednisolone 5 mg, which is no longer recommended, if cetirizine alone did not adequately control SAR symptoms) were provided on demand as RMs for both groups. No other RM was used.

### Outcome measurements

After the baseline assessment, outcomes were measured after 4 and 8 weeks with standardized questionnaires (RQLQ, Short Form-36 [SF-36], Trait-Havelhöher Konstitutionsfragebogen [T-HKF]) and weekly with standardized patient diaries (RMS, RQLQ week 2, VAS overall SAR symptoms, Total Nasal Symptom Severity [TNSS] and Total Non-Nasal Symptom severity [TNNSS]) (Additional file 1).

The validated RQLQ assesses disease-specific quality of life, including functional (physical, emotional and social) problems that are troublesome to adults with SAR, on a range of 0–6 points. The RQLQ contains seven domains (activity limitation, sleep problems, general discomforts, practical problems, nose and eye symptoms, and emotional function) with 28 items that are equally weighted and expressed as a score ranked from 0 (no impairment) to 6 (severe impairment). Lower values indicate better status. An average change in RQLQ score of 0.5 per domain and for the total score has been defined as the minimum clinically important difference (MCID) [27].

We rated the intake of anti-allergic drugs using the validated RMS (range 0–3) from weeks 1 to 4 and in week 8. The RMS comprises the daily mean SAR medication usage, in which the medication is evaluated by a point system ranging from 0 to 3 (no rhinitis medication [0 points]; cetirizine, 10 mg/d [1 point]; cetirizine, 20 mg/d [2 points]; oral steroid for SAR [3 points]). The point value of each day is represented only by the drug with the highest point value [28, 29].

Overall SAR symptom severity was assessed and rated on a validated VAS (0 mm = no symptoms to 100 mm = worst symptoms) [30]. Based on a strong correlation between the RQLQ and the VAS and a corresponding RQLQ MCID of 0.5 points, a change of 23 mm in the VAS was considered clinically important [31].

To assess the severity of patients’ nasal and non-nasal symptoms, we evaluated four nasal symptoms (sneezing, rhinorrhoea, nasal congestion, and nasal itching) and four non-nasal symptoms (eye itching, watery eyes, palatine itching, ear itching) using a four-point Likert scale that ranged from “no symptoms” [0 points] to “severe symptoms” [3 points]. Values were summed up to a maximum score of 12 points for the TNSS resp. TNNSS [32]. According to Meltzer et al., a threshold of 3.6 points on a TNSS scale of 0–12 or a 30% difference in maximum TNSS change from baseline was recommended by the Agency for Healthcare Research and Quality to define an MCID [33]. The German version has not been validated.

The validated T-HKF consists of 18 items and three subscales (orthostatic-circulatory, rest/activity and digestive regulation), allowing the evaluation of autonomous functions (e.g., vertigo, thermoregulation), including chronobiological aspects. The results range from 18 points (low autonomous regulation) to 54 points (high autonomous regulation). Higher values indicate less impairment [34].

To assess the validated health-related quality of life, we used the SF-36 (range 0–100, MCID 5 points) [35]. It consists of 36 questions with two sum scores (physical and mental) and eight health-related domains (vitality, physical function, physical pain, role [physical and emotional], social functioning, mental well-being, and general health perception). Higher values indicate better quality of life [36].

#### Further assessments

Therapy-related adverse events (AEs) and the feasibility, intensity and frequency of acupressure were assessed with patients’ diaries during the first 4 weeks and at week 8. In addition, standardized questions about the acupressure modality (which points, frequency, etc.), perceived effectiveness of acupressure regarding SAR, feasibility, safety and the potential need for further training were included in the 5- to 10-min telephone interviews after weeks 1 and 3.

To assess the health-economic aspect of acupressure, we investigated the direct costs due to RM use between weeks 1 and 4 in both groups as an additional outcome. The self-reported amount of RM use was monetarily assessed by using net cost per daily dose published by the Scientific Institute of the AOK (WIdO) [37].

### Statistical analysis

We determined the sample size primarily by considering logistics. We assumed that 20–30 patients per group (40–60 patients in total) were adequate to assess the feasibility and preliminary effect estimates of the acupressure. This sample size allowed a post hoc determination of moderate to large effects (effect size [Cohen’s d] 0.65–0.75, alpha 5%, 2-sided, assuming a power of 80% and performing a t-test) for the RQLQ.

Analysis was carried out based on the full analysis set (FAS) following an intention-to-treat principle with all randomized patients. Patients were evaluated according to their randomization assignment (independent of the performed acupressure). Missing data were not replaced. All data were analysed descriptively for each group and the whole study population. Analysis of covariance (ANCOVA) was used to compare continuous groups on follow-up measures with the respective baseline value, if existing, as a covariate. The results from ANCOVA are presented as adjusted means, 95% CIs and 2-sided p-values for group differences. Confidence intervals and (2-sided) p-values were interpreted exploratively. Binary outcomes (RQLQ responder analysis) were analysed using logistic regression with the baseline value as a covariate. The outcome RMS was evaluated descriptively due to the special measurement structure. In a post hoc analysis, we calculated the effect size based on adjusted means (Cohen’s d) for the RQLQ outcome after week 4 [38]. Since no baseline value existed to adjust for RM costs, an unpaired t-test analysis without baseline adjustment was performed for group comparisons. Due to the skewed distribution of cost variables, the corresponding p-value was based on 1000 mean bootstrap samples. Statistical analysis was performed with R (version 3.6.3) [39] and SPSS (version 26).

## Results

**Fig. 1:**
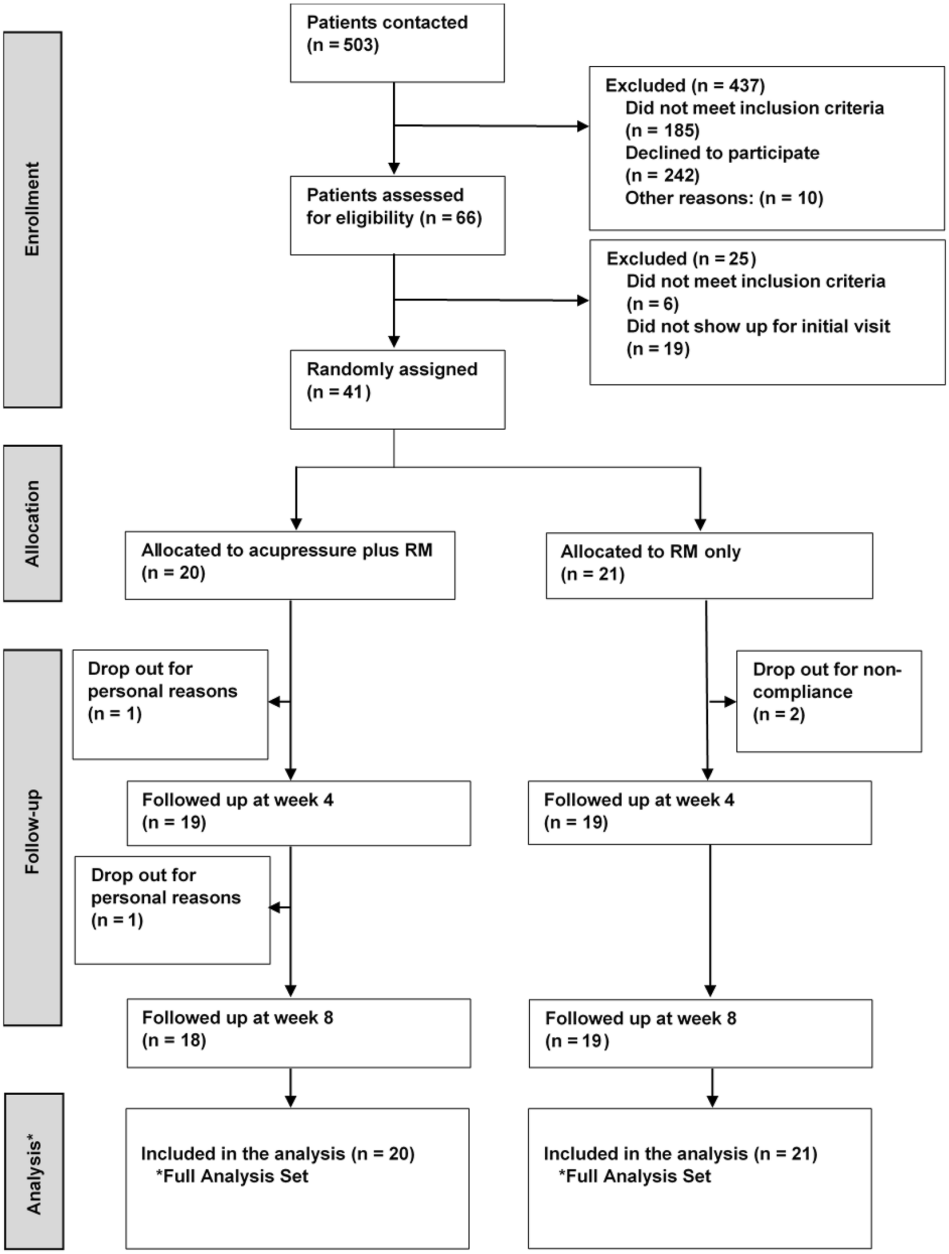
Study flow chart

In total, 503 SAR patients were screened by telephone by the study staff; 66 met the criteria for an initial visit. After carefully checking the patients to ensure they met the inclusion and exclusion criteria, 41 patients were enrolled (n = 14 between April and June in 2018; n = 27 between April and July in 2019) and assigned to the acupressure group (n = 20) or the control group (n = 21). We registered four dropouts after inclusion in the study: two in the acupressure group (one in week 4 and one in week 8) and two in the control group before week 4 (Fig. 1).

Overall, baseline characteristics were comparable between groups. Fifty-one percent of the study population (n = 21 [n = 10 acupressure group, n = 11 control group]) had used anti-allergic medication over 14 days prior to baseline. Antihistamines were used most frequently (71%, n = 15 [n = 9 acupressure group, n = 6 control group]), followed by cromoglicic acid (14%, n = 3 [n = 2 acupressure group, n = 1 control group]) and nasal cortisone spray (n = 1; control group). None of the patients had used oral corticoids. Baseline data for the RQLQ, VAS overall SAR symptoms, TNSS and TNNSS showed comparable severity in both groups (Table 2).

**Table 2:** Baseline characteristics

	All patients (n = 41)	Acupressure (n = 20)	Control (n = 21)
Mean age (SD), y	38.5 (10.0)	38.6 (10.6)	38.4 (9.7)
Female, n (%)	21.0 (51.2)	9.0 (45.0)	12.0 (57.1)
Mean BMI (SD), kg/m^2^	23.6 (4.2)	24.1 (4.7)	23.2 (3.7)
Mean duration of SAR (SD), y	21.2 (9.6)	22.8 (10.0)	19.7 (9.2)
Mean time since first diagnosis (SD), y	16.6 (9.6)	16.6 (9.6)	16.6 (9.9)
Allergy			
To birch and grass only, n (%)	12 (29.3)	9 (45.0)	3 (14.3)
To birch, grass and others, n (%)	29 (70.7)	11 (55.0)	18 (85.7)
Confirmation of allergy			
Prick test, n (%)	39 (95.1)	19 (95.0)	20 (95.2)
IgE/RAST test, n (%)	4 (9.8)	1 (5.0)	3 (14.3)
Therapy			
Prior desensitisation, n (%)	23 (56.1)	14 (70.0)	9 (42.9)
CAM use ever before, n (%)	19 (46.3)	11 (55.0)	8 (30.1)
Prior acupuncture treatment, n (%)	20 (48.8)	10 (50.0)	10 (47.6)
For SAR, n (%)	7 (17.0)	2 (10.0)	5 (23.8)
For other diagnosis, n (%)	13 (31.7)	8 (40.0)	5 (23.8)
Prior acupressure treatment, n (%)	3 (7.3)	2 (10.0)	1 (4.8)
For SAR, n (%)	0 (0.0)	0 (0.0)	0 (0.0)
For other diagnosis, n (%)	3 (7.3)	2 (10.0)	1 (4.8)
Outcome parameters			
Mean RQLQ overall score (SD)^a^	2.4 (0.9)	2.5 (0.8)	2.3 (1.0)
Mean VAS score (SD), mm^a^	51.1 (12.2)	50.5 (11.3)	51.6 (13.3)
Symptom severity score			
Mean TNSS/nasal score (SD)^a^	7.1 (2.1)	7.1 (2.1)	7.1 (2.2)
Mean TNNSS/non-nasal score (SD)^a^	3.7 (2.3)	4.1 (2.8)	3.3 (1.7)
Mean SF-36 score			
Physical health, mean (SD)^b^	49.3 (6.3)	48.7 (5.8)	49 (6.8)
Mental health, mean (SD)^b^	47.9 (8.1)	47.0 (7.7)	48.8 (8.7)
Mean T-HKF score			
Autonomic functioning, total, mean (SD)	41.8 (3.9)	40.5 (4.1)	43 (3.3)
Acupressure considered effective, n (%)	35 (75.0)	17 (85.0)	18 (85.7)
Expectation of significant recovery, n (%)	19 (46.3)	10 (50.0)	9 (42.9)
Years of recruitment, n (%)			
2018, n (%)	14 (34.1)	4 (20.0)	10 (47.6)
2019, n (%)	27 (65.9)	16 (80.0)	11 (55.0)

*BMI* body mass index, *CAM* complementary and alternative medicine, *RQLQ* Rhinitis Quality of Life Questionnaire, *SAR* seasonal allergic rhinitis, *SF-36* Short form-36 Health Survey, *VAS* visual analog scale for overall SAR symptoms, *TNSS* total nasal symptom severity, *TNNSS* total non-nasal symptom severity, *y* years

^a^Lower value indicates better status

^b^Higher value indicates better status

Acupressure was shown to be easily carried out and integrated into daily routines after a thorough introduction and training. Most patients (68%) preferred a single 20-min session daily, whereas 32% applied two or more sessions of 10 min daily. The individual daily acupressure duration was 16 min on average (16.9 min in week 1 and 16.1 min in week 4). Altogether, 9 patients (47%) reported initial problems and difficulties regarding the acupressure technique.

**Fig. 2:**
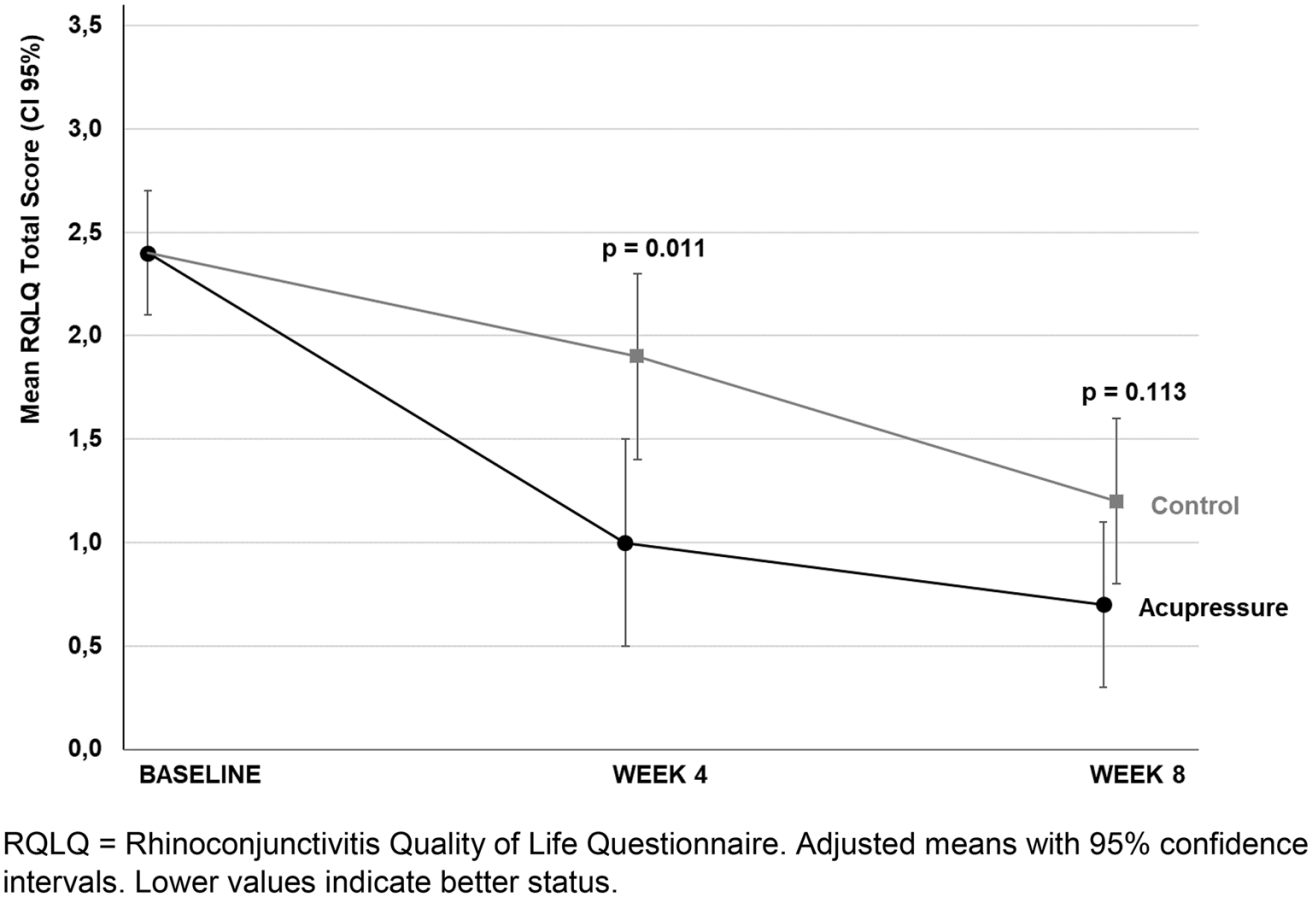
RQLQ Total Score at baseline, in weeks 4 and 8. *RQLQ* Rhinoconjunctivitis Quality of Life Questionnaire. Adjusted means with 95% confidence intervals. Lower values indicate better status

**Fig. 3:**
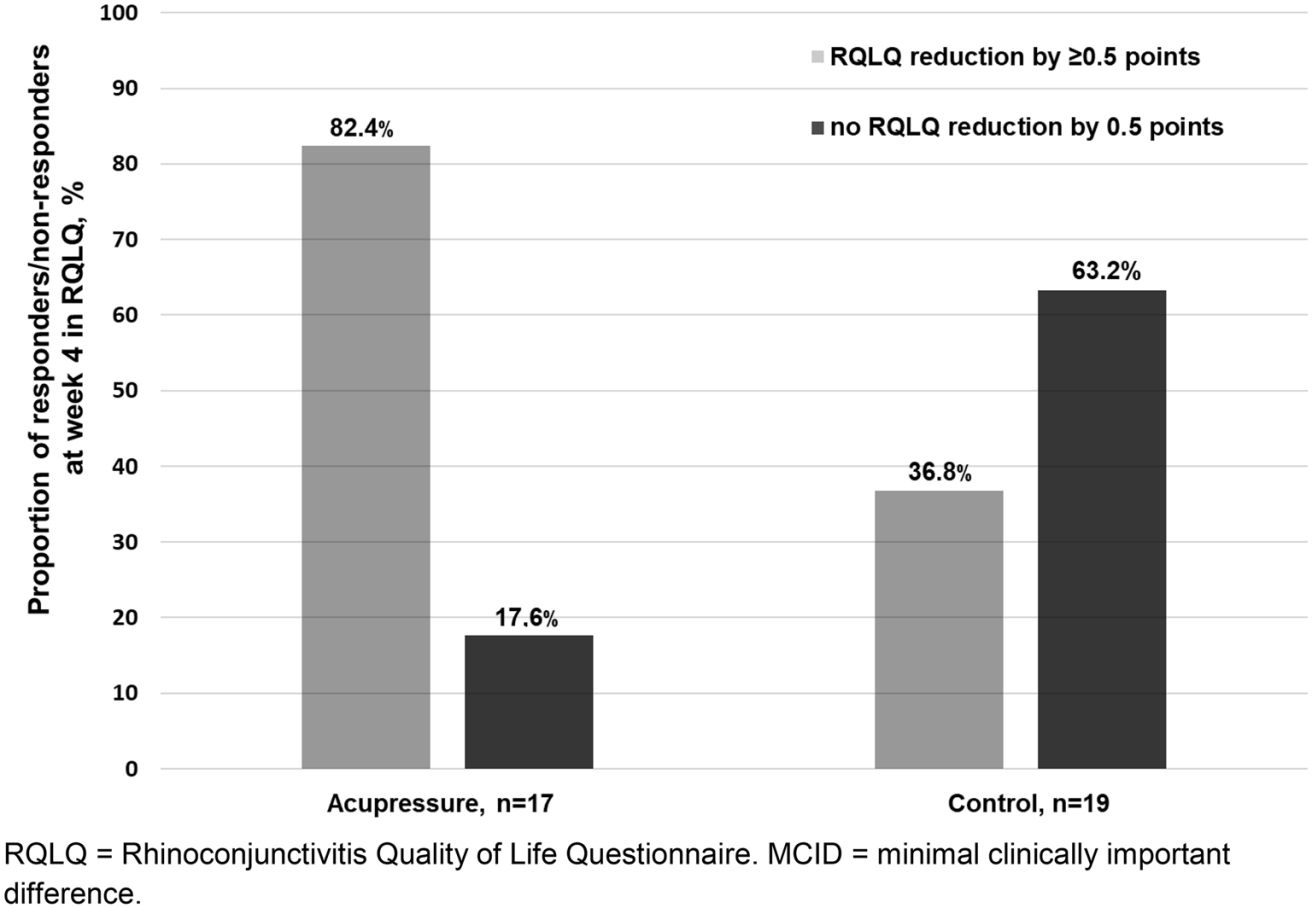
RQLQ responder vs. non-responder in week 4 (reduction of RQLQ Score at a MCID of 0.5 points). *RQLQ* Rhinoconjunctivitis Quality of Life Questionnaire, *MCID* minimal clinically important difference

To harmonize the way of reporting and for clarity, we decided to only show values at baseline, in weeks 4 and 8 in the figures. We found a noticeable decrease in the RQLQ total score in both groups over the study period (Table 3 and Fig. 2). However, after week 4, the group difference in adjusted means reached 0.9 points (CI 95%, − 1.6 to − 0.2; p = 0.011) in favour of the acupressure group and thus reached the MCID of 0.5 points. The RQLQ responder rates reached 82.4% in the acupressure group versus 36.8% in the control group (Fig. 3). The effect size for the RQLQ total score based on adjusted means showed overall high values [d = 0.9] (Table 3). The highest effect sizes after 4 weeks were found for the RQLQ domains sleep [d = 1.1], emotional function [d = 1.0] and nasal score [d = 0.9].

**Table 3:** Outcome measurements

Outcome	N^a^	Adjusted means (95% CI)			*P* value
		Acupressure	Control	Difference between groups Acupressure vs. control	
RQLQ total score (0–6)^b^ (MCID 0.5)					
After 2 weeks	38	1.4 (1.1 to 1.7)	1.7 (1.4 to 2.0)	− 0.3 (− 0.8 to 0.1)	0.133
After 4 weeks	36	1.0 (0.5 to 1.5)	1.9 (1.4 to 2.3)	− 0.9 (− 1.6 to − 0.2)	0.011
After 8 weeks	35	0.7 (0.3 to 1.1)	1.2 (0.8 to 1.6)	− 0.5 (− 1.1 to 0.1)	0.113
RMS (0–3)^b,d^					
After 4 weeks	37	0.1 (− 1.1 to 1.3)	2.0 (0.7 to 3.3)	− 1.9 (− 3.8 to − 0.1)	0.120
After 8 weeks	35	0.0 (− 1.5 to 2.0)	1.8 (0.4 to 3.0)	− 1.8 (− 4.0 to 0.3)	0.080
VAS score (0–100) mm^b^					
After 2 weeks	38	36.5 (27.2 to 45.9)	39.0 (29.7 to 48.3)	− 2.5 (− 15.7 to 10.7)	0.708
After 4 weeks	38	20.0 (9.6 to 30.4)	41.6 (31.2 to 52.0)	− 21.6 (− 36.3 to − 6.8)	0.005
After 8 weeks	33	16.7 (5.0 to 28.4)	31.3 (19.3 to 43.4)	− 14.6 (− 31.4 to 2.1)	0.085
TNSS (0–12)^b^					
After 2 weeks	38	4.3 (3.3 to 5.3)	5.2 (4.2 to 6.2)	− 0.8 (− 2.2 to 0.6)	0.231
After 4 weeks	38	3.0 (2.0 to 4.0)	5.3 (4.2 to 6.3)	− 2.3 (− 3.7 to − 0.8)	0.003
After 8 weeks	34	2.1 (0.9 to 3.3)	4.1 (3.0 to 5.3)	− 2.0 (− 3.7 to − 0.4)	0.019
TNNSS (0–12)^b^					
After 2 weeks	38	1.7 (1.0 to 2.5)	2.7 (2.0 to 3.5)	− 1.0 (− 2.1 to 0.1)	0.076
After 4 weeks	38	1.2 (0.3 to 2.1)	2.6 (1.8 to 3.5)	− 1.4 (− 2.6 to − 0.2)	0.026
After 8 weeks	33	1.1 (0.2 to 1.9)	1.9 (1.1 to 2.7)	− 0.9 (− 2.0 to 0.3)	0.137
THK-F (18–54)^c^ autonomic functioning					
After 4 weeks	36	41.5 (40.2 to 42.8)	40.9 (39.7 to 42.1)	0.6 (− 1.2 to 2.4)	0.505
After 8 weeks	35	41.9 (40.6 to 43.3)	41.3 (40.0 to 42.7)	0.6 (− 1.3 to 2.6)	0.527
SF-36 (0–100)^c^ Phys. component					
After 4 weeks	36	55.5 (52.8 to 58.1)	51.7 (49.2 to 54.2)	3.8 (0.2 to 7.4)	0.041
After 8 weeks	35	53.9 (51.7 to 56.0)	53.5 (51.4 to 55.6)	0.3 (− 2.7 to 3.4)	0.818
SF-36 (0–100)^c^ mental component					
After 4 weeks	36	47.2 (42.8 to 51.6)	48.6 (44.4 to 52.7)	− 1.4 (− 7.5 to 4.7)	0.649
After 8 weeks	35	48.7 (44.6 to 52.8)	50.8 (46.8 to 54.8)	− 2.1 (− 7.9 to 3.6)	0.457

Outcome	N^a^	RQLQ-related Outcomes			
		Acupressure	Control	Difference between groups Acupressure vs. control	
RQLQ responder rates (%)^c^					
After week 4	36	82.4	36.8	45.6	
RQLQ effect size^c,e^					
After week 4	36		0.9		
After week 8	35		0.6		

*RQLQ* Rhinitis Quality of Life Questionnaire, *RMS* rescue medication score, non-adjusted means, *VAS* visual analog scale for overall SAR symptoms, *TNSS* total nasal symptom score, *TNNSS* total non-nasal symptom score, *THK-F* trait-constitution questionnaire of Havelhöhe, *SF-36* Short form-36 Health Survey, *MCID* minimal clinically important difference

^a^Number shown is the number of randomly assigned patients, analysed numbers vary because of missings

^b^Lower values indicate better status

^c^Higher values indicate better status

^d^Without baseline-adjustment

^e^Effect size based on adjusted means

**Fig. 4:**
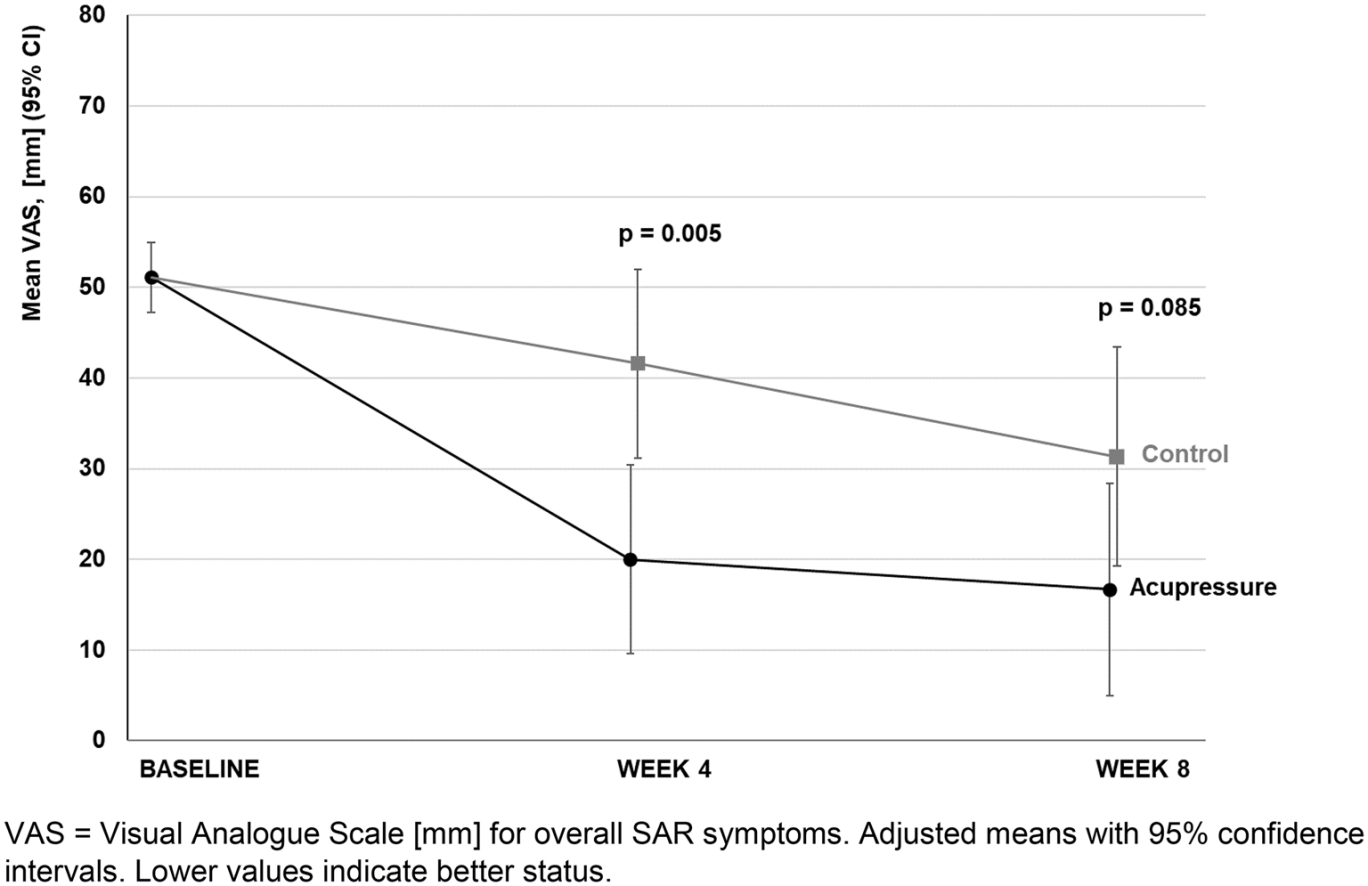
VAS overall SAR symptoms at baseline, in weeks 4 and 8. *VAS* Visual Analogue Scale (mm) for overall SAR symptoms. Adjusted means with 95% confidence intervals. Lower values indicate better status

**Fig. 5:**
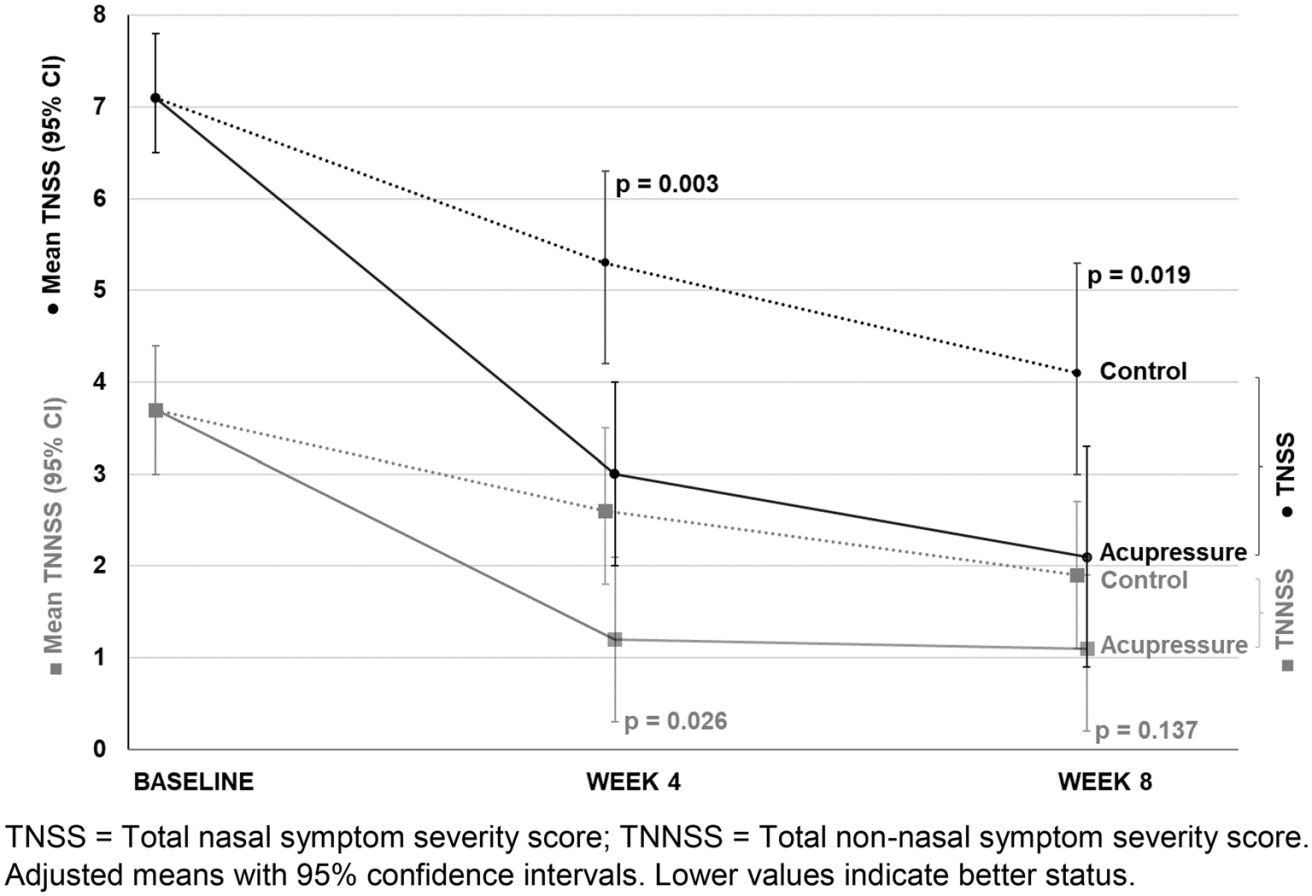
TNSS and TNNSS at baseline, in weeks 4 and 8. *TNSS* total nasal symptom severity score, *TNNSS* total non-nasal symptom severity score. Adjusted means with 95% confidence intervals. Lower values indicate better status

The VAS for overall SAR symptoms, the TNSS and the TNNSS showed lower values in the acupressure group than the control group and highlighted differences between groups in favour of the acupressure group after week 4: VAS − 21.6 mm (95% CI − 36.3 to − 6.8; p = 0.005); TNSS − 2.3 points (95% CI − 3.7 to − 0.8; p = 0.003); and TNNSS -1.4 points (CI 95% − 2.6 to − 0.2; p = 0.026) (Table 3 and Figs. 4, 5). The T-HKF total score and SF-36 showed only minor or no differences between groups (Table 3).

After week 4, the acupressure group showed a lower RMS than the control group (mean RMS 0.1 ± 0.3 vs. 2.0 ± 3.8, group difference of adjusted means of 1.9 points, CI 95% − 3.8 to − 0.1; p = 0.120) (Table 3)*.* During the 4-week intervention period, RM was used by 36% (week 1) to 11% (week 4) of the patients in the acupressure group and by 47% (week 1) to 37% (week 4) of the patients in the control group. In week 8, none of the patients in the acupressure group used RM, while 22% of the patients in the control group still did.

After 4 weeks, 76% of the patients considered the acupressure “effective” or “very effective”, and 88% were very satisfied or satisfied with the acupressure. Those estimations persisted until week 8.

No serious AEs were observed during the entire study period. According to the patients’ diaries, 3 (16%) patients in the acupressure group recorded therapy-related minor AEs (nausea [n = 2] and mild pain at the acupressure points [n = 3]), which did not necessitate therapeutic intervention.

Although RM is well established and comparably inexpensive, the health-economic analysis of the direct weekly costs of RM showed noticeable differences between groups. Over the course of the study, we assessed mean direct costs of 0.14 € per week/per person in the acupressure group and 0.90 € per week/per person in the control group (p = 0.044; 1.05 € vs. 0.34 € in week 1, 1.14 € vs. 0.04€ in week 4 and 0.52 € vs. 0.00 € in week 8, in favour of the acupressure group).

## Discussion

The results of this exploratory study indicate that self-applied acupressure is feasible and may improve disease-specific quality of life and reduce disease-related symptoms as well as anti-allergic medication intake in SAR patients. In addition, self-applied acupressure showed high overall adherence and a good safety profile.

To the best of our knowledge, our study is the first exploratory RCT on SAR that compares self-applied acupressure to RM alone using outcome parameters that have been utilized in previous large high-quality trials on acupuncture in SAR [13, 22, 40]. The strength of this trial included an adapted consensus Delphi approach with CM experts to select acupressure points that were highly standardized in regard to the location, duration, frequency and intensity of the acupressure intervention. In addition, acupressure was easily applicable, as only five points were used, and patients were able to apply acupressure regardless of their location. Patients were instructed and motivated thoroughly through a teaching session, a manual session and a DVD with details on the acupressure technique. Throughout patients’ diaries, telephone calls and questionnaires, we ensured adherence to the intervention by assessing the correct acupressure modality frequently and systematically. Patient compliance was high, and overall AEs remained low. Allowing the patients a self-treatment saved therapeutic costs [4, 41] and certainly strengthened their self-efficacy and autonomy, which may be a crucial effect of the intervention [42].

The main limitation of this exploratory trial is the use of a waiting list design, with the use of RM in both groups but a lack of a control intervention. Furthermore, the difference in doctor-patient interaction in terms of frequency and time (two telephone calls and an approximately one-hour longer contact in the acupressure group) between the groups entails a limitation for the impact on the results. Thus, these aspects should be balanced between groups when planning confirmative trials with active or sham controls [43]. In addition, we did not establish blinding in our study, which would have further reduced the risk of bias, e.g., performance bias. A single blinding would have been possible with an adequate sham procedure at non-acupuncture points or with using acupressure devices (e.g. bands) which apply pressure with a predetermined force instead of manual pressure [44–46]. A double-blind protocol may be realizable using only videos to demonstrate the real and sham acupressure points, however the study doctor’ control of the acupressure technique and assurance of safety would be limited. Therefore, the acupressure procedure in our study could only be standardized and controlled to a certain extent since it was self-applied by the patients at home. Finally, to minimize possible improper operations and reduce the risk of excessive pressure, an acupressure device with a pressure sensor showing the exact acupressure applied could be used [45, 46].

To date, research on the specific effects of acupressure is limited [47], while for acupuncture, it is assumed that only approximately one-third of the effect is due to specific needle location and needling techniques (e.g., “de-qi” feeling), and two-thirds of the effects are nonspecific (e.g., physiological effects due to skin contact and psychological effects due to increased awareness, continuity, beliefs in the treatment, doctor-patient relationships) [48–50]. Assuming that there are also nonspecific effects of acupressure due to the grade of attention, the doctor–patient time and positive expectations regarding acupressure, we can speculate that unspecific effects played an important role in our study and therefore affected our results to a certain extent. Because we only assessed patient-related outcome parameters, which are highly subjective and subject to placebo effects, our results are potentially unreliable to a certain extent [51]. Nevertheless, “placebo effects can be clinically meaningful” [51] and therefore crucial for improving patients’ quality of life.

Because scientific evidence for acupressure in SAR is limited [22], we compared acupressure to larger trials on acupuncture, such as the ACUSAR trial. This trial was a three-armed RCT (n = 422; acupuncture plus RM [n = 212], sham acupuncture plus RM [n = 102] or RM alone [n = 108]) that assessed the effects of 12 acupuncture sessions on disease-related quality of life and symptoms as well as RM use in SAR patients for 8 weeks [13]. While baseline values for the RQLQ and VAS overall SAR symptoms were comparable between ACUSAR and our study, we found much lower RQLQ values for acupressure in our study than acupuncture in ACUSAR at week 8, while group differences within the studies (acupressure versus control, acupuncture versus control) remained comparable. A meta-analysis of 13 trials on SAR and perennial allergic rhinitis with acupuncture interventions of 4 to 10 weeks duration (n = 2365) as well as a recent two-armed RCT (n = 175) on 12 acupressure sessions over 4 weeks in SAR patients showed RQLQ values comparable to our results regarding differences between groups when compared to standard care or waiting lists [12, 52]. Results of a large 3-armed pragmatic partly randomized trial in 5237 allergic rhinitis patients compared two randomized arms including acupuncture plus routine care (n = 487) and routine care only (n = 494) to one non-randomized arm with acupuncture plus routine care (n = 4256) and showed comparable response rates for disease-specific quality of life to acupressure after 3 months as found in our study after 2 months. Group differences in nasal and non-nasal symptom severity scores were most noticeable after week 4 and were comparable to a three-armed multicentre RCT in 238 patients, including active acupuncture, sham acupuncture and a waiting list control on acupuncture in allergic rhinitis patients [40].

In our study, nasal symptoms improved more under acupressure than non-nasal symptoms, which may be related to the choice of acupressure points. While LI 20 is indicated and often used to relieve nasal symptoms in acupuncture, Ex-HN 3 (Yintang) is also used to treat discomfort of the nose and, less specifically, to treat eye discomfort in general [23]. In addition to the effects within the CM system (mobilization of qi, expelling of wind, immune modulating and anti-inflammatory effects) [23], potential mechanisms of action such as reduction of pathological airway remodelling, downregulation of H1 and H4 receptor proteins and analgesic effects have been recently shown for acupoints LI 4, LI20, Ex-HN 3 (Yintang) and Gb20 [24–26, 53]. The greater decrease in RM intake in the acupressure group may be due to a stronger symptom alleviation through the acupressure or due to the possible motivation in the acupressure group to address symptoms with acupressure rather than with anti-allergic drugs. However, reduced RM intake decreases the risk of potential anti-allergic drug-related AEs [54], which could have a considerable positive impact on disease-related quality of life.

Although the daily costs of anti-allergic drugs (RM) in our study remained generally low, the costs of RM in the acupressure group were six times lower than those in the control group, which demonstrates the health-economic potential of acupressure in SAR.

To date, the physiology and mechanism of the effect of acupressure are largely unexplored, whereas the possible effects of acupuncture within the central nervous system and allergen response on a molecular level have been demonstrated in several studies [55–58]. Research on the structure of acupuncture points (APs) indicates a strong concentration of terminal nerve fibres of type Aβ, Aδ and C at the sites of an AP or along the meridians [59], whose stimulation could trigger neurological and molecular responses [60–62] and may mediate the expression of inflammatory cytokines and neuropeptides [63]. For acupuncture, it is assumed that needle rotation leads to the stretching of fibroblasts, which triggers mechano-sensory signal transduction, resulting in neuromodulation [64]. Acupressure does not penetrate the skin, but as it has a mechanical impact on a larger area, it might stimulate all three fibres, particularly the free ending Aδ fibres, which are cold and pressure sensitive, and the C fibres, which are sensitive to heat and responsive to histamine-induced itch [65]. Stimulating these nerve endings could lead to pain inhibition mechanisms and reduce itching, the main symptom in SAR [61]. Studies have shown that acupressure can modulate blood circulation on the body surface and significantly increase regional oxygen saturation compared to acupressure on non-acupoints [66, 67]. Taken together, we assume that the mechanisms of acupressure might pertain to immunological and neurological functions [61]. However, further experimental research is required to understand the underlying mechanisms of acupressure.

## Conclusion

The results of this exploratory study indicate that self-applied acupressure is feasible and may improve disease-specific quality of life and reduce disease-related symptoms as well as anti-allergic medication intake in SAR patients. In addition, acupressure was relatively safe and reduced costs for anti-allergic medications. The overall and specific effects of acupressure in SAR should be investigated in further high-quality confirmatory studies that include a sham-control group.

## Supplementary Information

**Additional file 1.** Study design and data assessment schedule.

## Data Availability

The datasets used and/or analysed during the current study are available from the corresponding author on reasonable request.

## Abbreviations

AEs: Adverse events
ANCOVA: Analysis of covariance
CM: Chinese medicine
Ex-HN 3: Extraordinary point
FAS: Full analysis set
LI-20: Large intestine-20
MCID: Minimal clinically important difference
MT: Massage therapy
RCT: Randomized controlled trial
RM: Rescue medication
RMS: Rescue Medication Score
RQLQ: Rhinitis Quality of Life Questionnaire
SAR: Seasonal allergic rhinitis
SF-36: Short Form-36
T-HKF: Trait-constitution questionnaire of Havelhöhe
TNSS: Total nasal symptom score
TNNSS: Total non-nasal symptom score

## References

[1] Long A, McFadden C, DeVine D, Chew P, Kupelnick B, Lau J (2002). Management of allergic and nonallergic rhinitis. Evid Rep Technol Assess (Summ).

[2] Bachert C, van Cauwenberge P, Olbrecht J, van Schoor J (2006). Prevalence, classification and perception of allergic and nonallergic rhinitis in Belgium. Allergy.

[3] Nathan RA, Meltzer EO, Derebery J, Campbell UB, Stang PE, Corrao MA (2008). The prevalence of nasal symptoms attributed to allergies in the United States: findings from the burden of rhinitis in an America survey. Allergy Asthma Proc.

[4] Blaiss MS (2010). Allergic rhinitis: direct and indirect costs. Allergy Asthma Proc.

[5] Muche-Borowski C, Kopp M, Reese I, Sitter H, Werfel T, Schäfer T (2010). Allergy prevention. J Dtsch Dermatol Ges.

[6] Klimek L, Bachert C, Pfaar O, Becker S, Bieber T, Brehler R (2019). ARIA guideline 2019: treatment of allergic rhinitis in the German health system. Allergo J Int.

[7] Seidman MD, Gurgel RK, Lin SY, Schwartz SR, Baroody FM, Bonner JR (2015). Clinical practice guideline: allergic rhinitis. Otolaryngol Head Neck Surg.

[8] Bousquet J, Anto JM, Bachert C, Baiardini I, Bosnic-Anticevich S, Walter Canonica G (2020). Allergic rhinitis. Nat Rev Dis Prim.

[9] Greiner AN, Hellings PW, Rotiroti G, Scadding GK (2011). Allergic rhinitis. Lancet.

[10] Krouse HJ, Krouse JH (1999). Complementary therapeutic practices in patients with chronic sinusitis. Clin Excell Nurse Pract.

[11] Schäfer T, Riehle A, Wichmann HE, Ring J (2002). Alternative medicine in allergies—prevalence, patterns of use, and costs. Allergy.

[12] Xue CC, Zhang AL, Zhang CS, DaCosta C, Story DF, Thien FC (2015). Acupuncture for seasonal allergic rhinitis: a randomized controlled trial. Ann Allergy Asthma Immunol.

[13] Brinkhaus B, Ortiz M, Witt CM, Roll S, Linde K, Pfab F (2013). Acupuncture in patients with seasonal allergic rhinitis: a randomized trial. Ann Intern Med.

[14] Beal MW (1999). Acupuncture and acupressure. Applications to women’s reproductive health care. J Nurse Midwifery.

[15] Boguszewski D, Krupiński M, Białoszewski D (2017). Assessment of the effect of Swedish massage and acupressure in rehabilitation of patients with low back pain. Preliminary report. Ortop Traumatol Rehabil.

[16] Shin W (2013). The effect of convalescent meridian acupressure after exercise on stress hormones and lactic acid concentration changes. J Exerc Rehabil.

[17] Zhang CS, Xia J, Zhang AL, Yang AW, Thien F, Li Y (2014). Ear acupressure for perennial allergic rhinitis: a multicenter randomized controlled trial. Am J Rhinol Allergy.

[18] Miao J, Liu X, Wu C, Kong H, Xie W, Liu K (2017). Effects of acupressure on chemotherapy-induced nausea and vomiting—a systematic review with meta-analyses and trial sequential analysis of randomized controlled trials. Int J Nurs Stud.

[19] Gharloghi S, Torkzahrani S, Akbarzadeh AR, Heshmat R (2012). The effects of acupressure on severity of primary dysmenorrhea. Patient Prefer Adherence.

[20] Zick SM, Alrawi S, Merel G, Burris B, Sen A, Litzinger A (2011). Relaxation acupressure reduces persistent cancer-related fatigue. Evid Based Complement Alternat Med.

[21] Smith CA, Armour M, Dahlen HG (2017). Acupuncture or acupressure for induction of labour. Cochrane Database Syst Rev.

[22] Liang Y, Lenon GB, Yang AWH (2017). Acupressure for respiratory allergic diseases: a systematic review of randomised controlled trials. Acupunct Med.

[23] Lian Y, Chen C, Hammes M, Kolster B (2013). Bildatlas der Akupunktur - Darstellung der Akupunkturpunkte.

[24] Maa SH, Wang CH, Hsu KH, Lin HC, Yee B, Macdonald K (2013). Acupressure improves the weaning indices of tidal volumes and rapid shallow breathing index in stable coma patients receiving mechanical ventilation: randomized controlled trial. Evid Based Complement Alternat Med.

[25] Liu JH, Gu JW, Hu Q, Yue GL, Liu L, Tu X (2020). Effect of catgut implantation at “Yingxiang” (LI20) on lower airway remodeling in allergic rhinitis rats. Zhen Ci Yan Jiu.

[26] Zhao L, Liu L, Xu X, Qu Z, Zhu Y, Li Z (2020). Electroacupuncture inhibits hyperalgesia by alleviating inflammatory factors in a rat model of migraine. J Pain Res.

[27] Juniper EF, Thompson AK, Ferrie PJ, Roberts JN (1999). Validation of the standardized version of the rhinoconjunctivitis quality of life questionnaire. J Allergy Clin Immunol.

[28] Kuehr J, Brauburger J, Zielen S, Schauer U, Kamin W, Von Berg A (2002). Efficacy of combination treatment with anti-IgE plus specific immunotherapy in polysensitized children and adolescents with seasonal allergic rhinitis. J Allergy Clin Immunol.

[29] Grouin JM, Vicaut E, Devillier P (2017). Comparison of scores associating symptoms and rescue medication use for evaluating the efficacy of allergy immunotherapy in seasonal allergic rhinoconjunctivitis: results from five trials. Clin Exp Allergy.

[30] Klimek L, Bergmann KC, Biedermann T, Bousquet J, Hellings P, Jung K (2017). Visual analogue scales (VAS): measuring instruments for the documentation of symptoms and therapy monitoring in cases of allergic rhinitis in everyday health care: position paper of the German Society of Allergology (AeDA) and the German Society of Allergy and Clinical Immunology (DGAKI), ENT section, in collaboration with the working group on clinical immunology, allergology and environmental medicine of the German Society of Otorhinolaryngology, Head and Neck Surgery (DGHNOKHC). Allergo J Int.

[31] Demoly P, Bousquet PJ, Mesbah K, Bousquet J, Devillier P (2013). Visual analogue scale in patients treated for allergic rhinitis: an observational prospective study in primary care: asthma and rhinitis. Clin Exp Allergy.

[32] Spector SL, Nicklas RA, Chapman JA, Bernstein IL, Berger WE, Blessing-Moore J (2003). Symptom severity assessment of allergic rhinitis: part 1. Ann Allergy Asthma Immunol.

[33] Meltzer EO, Wallace D, Dykewicz M, Shneyer L (2016). Minimal clinically important difference (MCID) in allergic rhinitis: agency for healthcare research and quality or anchor-based thresholds?. J Allergy Clin Immunol Pract.

[34] Kröz M, Reif M, Pranga D, Zerm R, Schad F, Baars EW (2016). The questionnaire on autonomic regulation: a useful concept for integrative medicine?. J Integr Med.

[35] Wyrwich KW, Tierney WM, Babu AN, Kroenke K, Wolinsky FD (2005). A comparison of clinically important differences in health-related quality of life for patients with chronic lung disease, asthma, or heart disease. Health Serv Res.

[36] Bullinger M (1995). German translation and psychometric testing of the SF-36 health survey: preliminary results from the IQOLA project. International quality of life assessment. Soc Sci Med.

[37] PharMaAnalyst Berlin: Wissenschaftliches Institut der AOK (WIdO); 2020.

[38] Olejnik S, Algina J (2000). Measures of effect size for comparative studies: applications, interpretations, and limitations. Contemp Educ Psychol.

[39] A language and environment for statistical computing Vienna. https://www.R-project.org/.

[40] Choi SM, Park JE, Li SS, Jung H, Zi M, Kim TH (2013). A multicenter, randomized, controlled trial testing the effects of acupuncture on allergic rhinitis. Allergy.

[41] Cardell LO, Olsson P, Andersson M, Welin KO, Svensson J, Tennvall GR (2016). TOTALL: high cost of allergic rhinitis-a national Swedish population-based questionnaire study. NPJ Prim Care Respir Med.

[42] Bashtian MH, Khadivzadeh T, Aval SB, Esmaily H (2018). Evaluation of acupressure effects on self-efficacy and pregnancy rate in infertile women under in vitro fertilization/intracytoplasmic sperm injection treatment: a randomized controlled trial. J Educ Health Promot.

[43] Tan JY, Suen LK, Wang T, Molassiotis A (2015). Sham acupressure controls used in randomized controlled trials: a systematic review and critique. PLoS ONE.

[44] Nordio M, Romanelli F (2008). Efficacy of wrists overnight compression (HT 7 point) on insomniacs: possible role of melatonin?. Minerva Med.

[45] Mehta P, Dhapte V, Kadam S, Dhapte V (2017). Contemporary acupressure therapy: adroit cure for painless recovery of therapeutic ailments. J Tradit Complement Med.

[46] Zhang S, Zhu Q, Zhan C, Cheng W, Mingfang X, Fang M (2020). Acupressure therapy and Liu Zi Jue Qigong for pulmonary function and quality of life in patients with severe novel coronavirus pneumonia (COVID-19): a study protocol for a randomized controlled trial. Trials.

[47] Liang Y, Lenon GB, Yang AWH (2019). Self-administered acupressure for allergic rhinitis: study protocol for a randomized, single-blind, non-specific controlled, parallel trial. Trials.

[48] Irnich D, Salih N, Offenbächer M, Fleckenstein J (2011). Is sham laser a valid control for acupuncture trials?. Evid Based Complement Alternat Med.

[49] Linde K, Witt CM, Streng A, Weidenhammer W, Wagenpfeil S, Brinkhaus B (2007). The impact of patient expectations on outcomes in four randomized controlled trials of acupuncture in patients with chronic pain. Pain.

[50] Paterson C, Britten N (2008). The patient’s experience of holistic care: insights from acupuncture research. Chronic Illn.

[51] Wechsler ME, Kelley JM, Boyd IO, Dutile S, Marigowda G, Kirsch I (2011). Active albuterol or placebo, sham acupuncture, or no intervention in asthma. N Engl J Med.

[52] Feng S, Han M, Fan Y, Yang G, Liao Z, Liao W (2015). Acupuncture for the treatment of allergic rhinitis: a systematic review and meta-analysis. Am J Rhinol Allergy.

[53] Liang FH, Hou XR, Li LH, Liang X, Lu YW, Yang H (2018). Acupoint Injection at “Yingxiang”(LI 20) and “Yintang”(GV 29) may relieve nasal allergic symptoms possibly by down-regulating expression of histamine receptor H 1 and H 4 in nasal mucosa of allergic rhinitis rats. Zhen Ci Yan Jiu.

[54] Church MK, Maurer M, Simons FE, Bindslev-Jensen C, van Cauwenberge P, Bousquet J (2010). Risk of first-generation H(1)-antihistamines: a GA(2)LEN position paper. Allergy.

[55] Pfab F, Athanasiadis GI, Huss-Marp J, Fuqin J, Heuser B, Cifuentes L (2011). Effect of acupuncture on allergen-induced basophil activation in patients with atopic eczema: a pilot trial. J Altern Complement Med.

[56] Pfab F, Hammes M, Bäcker M, Huss-Marp J, Athanasiadis GI, Tölle TR (2005). Preventive effect of acupuncture on histamine-induced itch: a blinded, randomized, placebo-controlled, crossover trial. J Allergy Clin Immunol.

[57] Carlsson CP, Wallengren J (2010). Therapeutic and experimental therapeutic studies on acupuncture and itch: review of the literature. J Eur Acad Dermatol Venereol.

[58] Zijlstra FJ, van den Berg-de LI, Huygen FJ, Klein J (2003). Anti-inflammatory actions of acupuncture. Mediat Inflamm.

[59] Li AH, Zhang JM, Xie YK (2004). Human acupuncture points mapped in rats are associated with excitable muscle/skin-nerve complexes with enriched nerve endings. Brain Res.

[60] Huang W, Pach D, Napadow V, Park K, Long X, Neumann J (2012). Characterizing acupuncture stimuli using brain imaging with FMRI—a systematic review and meta-analysis of the literature. PLoS ONE.

[61] Irnich D, Beyer A (2002). Neurobiological mechanisms of acupuncture analgesia. Schmerz.

[62] Harris RE, Zubieta JK, Scott DJ, Napadow V, Gracely RH, Clauw DJ (2009). Traditional Chinese acupuncture and placebo (sham) acupuncture are differentiated by their effects on mu-opioid receptors (MORs). Neuroimage.

[63] Lin WC, Yeh CH, Chien LC, Morone NE, Glick RM, Albers KM (2015). The anti-inflammatory actions of auricular point acupressure for chronic low back pain. Evid Based Complement Alternat Med.

[64] Langevin HM, Bouffard NA, Badger GJ, Churchill DL, Howe AK (2006). Subcutaneous tissue fibroblast cytoskeletal remodeling induced by acupuncture: evidence for a mechanotransduction-based mechanism. J Cell Physiol.

[65] Gekle M, Wischmeyer E, Gründer S (2010). Taschenlehrbuch Physiologie.

[66] Hsiu H, Hsu WC, Chen BH, Hsu CL (2010). Differences in the microcirculatory effects of local skin surface contact pressure stimulation between acupoints and nonacupoints: possible relevance to acupressure. Physiol Meas.

[67] Litscher G, Ofner M, He W, Wang L, Gaischek I (2013). Acupressure at the Meridian Acupoint Xiyangguan (GB33) influences near-infrared spectroscopic parameters (regional oxygen saturation) in deeper tissue of the knee in healthy volunteers. Evid Based Complement Alternat Med.

