# Supplementary material for: Acupressure in patients with seasonal allergic rhinitis: a randomized controlled exploratory trial
Source: Chin Med. 2021 Dec 18;16:137. doi: 10.1186/s13020-021-00536-w (PMC8684198; doi:10.1186/s13020-021-00536-w)
Supplement: Supplementary file 1 — Additional file 1. Study design and data assessment schedule. [file 13020_2021_536_MOESM1_ESM.docx]

Additional file 1. **Study design and data assessment schedule.**


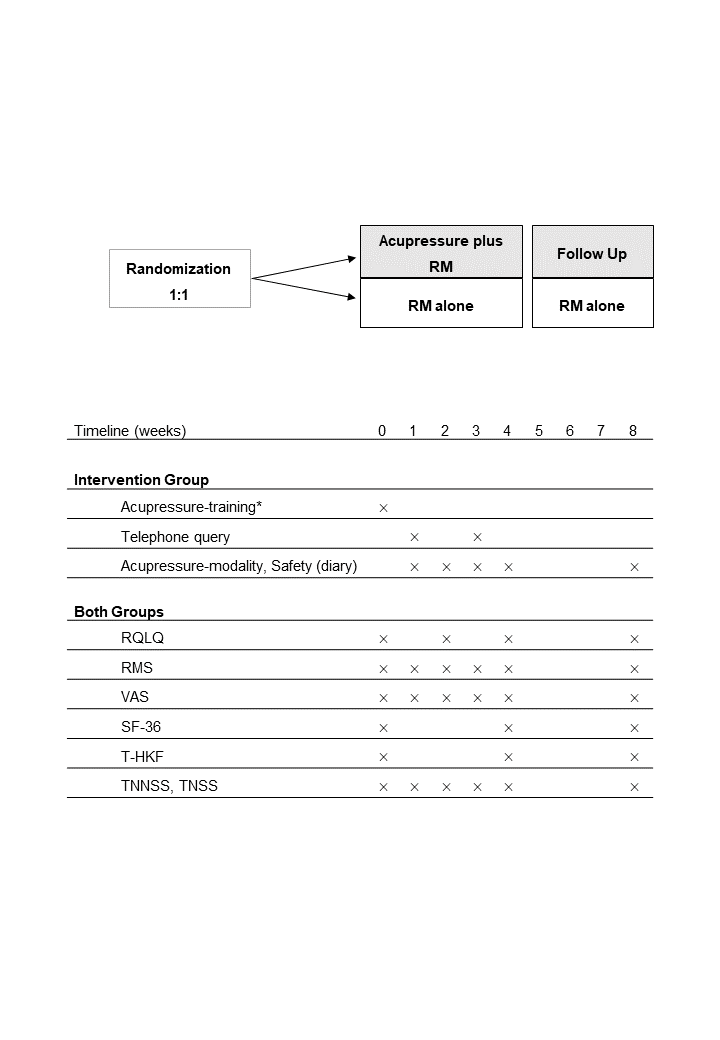


| **repeated training if needed, for Control Group: optional training after 8 weeks* |
| --- |
| RM = Rescue Medication, RQLQ = Rhinitis Quality of Life Questionnaire, RMS = Rescue Medication Score, VAS = Visual Analog Scale for overall SAR symptoms, SF-36 = Short Form-36 Health Survey, T-HKF = trait-constitution questionnaire of Havelhöhe, TNNSS, TNSS = total non-nasal & nasal SAR symptoms |
